# The Association Between Gait Speed and Sleep Problems Among Chinese Adults Aged 50 and Greater

**DOI:** 10.3389/fnins.2022.855955

**Published:** 2022-04-26

**Authors:** Lili Wang, Benxu Zou

**Affiliations:** ^1^School of Martial Arts and Dance, Shenyang Sport University, Shenyang, China; ^2^School of Social Sports, Shenyang Sport University, Shenyang, China

**Keywords:** sleep quality, physical function, gait speed, Chinese adults, walking speed

## Abstract

**Objective:**

The relationship between sleep problems and walking speed has been less explored. The present cross-sectional study was to investigate the association between sleep quality and sleep duration and gait speed in Chinese adults.

**Methods:**

A total of 13,367 participants were recruited in this cross-sectional study, retrieving the data from the Global Aging and Adult Health Survey (SAGE). Gait speed was measured using the 4-m walking test. Age, sex, education years, smoking status, alcohol consumption, physical activity, chronic disease, sleep problems were self-reported by participants. To explore the association between sleep problems and gait speed, multivariate linear regression models were employed.

**Results:**

In the adjusted model, poor sleep quality and longer sleep duration were significantly associated with slower normal walking speed in Chinese adults (*p* < 0.001). Moreover, there were negatively significant associations between normal gait speed and sleep quality in male adults (*p* < 0.01).

**Conclusion:**

The findings suggest that slower normal walking speed was associated with poor sleep quality and longer sleep duration (>8 h) in Chinese male adults.

## Introduction

Sleep problems are conditions that involve disturbance of normal sleep patterns. It has become an important public health issue in modern society, seriously affecting human health (Vega et al., 2019). Specifically, the typical symptoms of sleep-related problems are poor sleep quality, difficulty falling asleep, waking up frequently during the night, waking up early in the morning, daytime tiredness, and/or longer sleep duration (Gulia and Kumar, 2018). Sleep problems in older adults have attracted considerable attention in recent years as its prevalence has been increased. Specifically, two epidemiologic studies have reported poor sleep quality among aging population, with a prevalence of sleep problems ranging from 7.6 to 37.8% (Koyanagi and Stickley, 2015; Wang et al., 2016). Moreover, sleep problems were reported to be associated with a series of detrimental outcomes such as poor quality of life (Hinz et al., 2017), risk of falls (Takada et al., 2018), hospitalization (Wesselius et al., 2018), chronic diseases (Gulia and Kumar, 2018), and mortality (Wesselius et al., 2018).

Meanwhile, decline in physical function is relatively common among older adults, affecting their health and wellbeing. Walking speed is an essential component of physical function and is a reliable measure to reflect the activity of daily living and functional capacity (Peters et al., 2013; Nakakubo et al., 2018). Walking ability relays on the function and interaction of skeletal muscle, central and peripheral nervous systems, and requires aerobic capacity, cardiorespiratory fitness, and energy production and transmission (Jee et al., 2019). A decline in walking speed has been found to be closely related to disability, loss of independence, and frailty (Lewis, 2015; Pamoukdjian et al., 2015). On the contrary, elevated walking speed contributes to the constructive changes in quality of life later (Peters et al., 2013). Therefore, these studies suggest that walking speed is a convenient and effective method to evaluate individuals’ health condition as a practical clinical measure (Kim et al., 2015).

Taken together, both sleep problems and walking speed can affect human health, therefore, it could be presumably expected to observe an interaction between two health-related variables (Umemura et al., 2021). However, this topic on interaction between sleep problems and walking speed has been less investigated. An early study of Agmon et al. (2016) indicated that older adults with poor sleep quality had a worse performance under dual tasks, implying the compromise of gait ability. Furthermore, Kasović et al. (2021) reported that participants who walked at a fast speed had significantly good sleep quality in emerging adults. Of note, Nakakubo et al. (2018) reported that excessive daytime sleepiness was associated with slower walking speed in Japanese younger and older adults. Other studies suggested a strong link between sleep and gait speed among general adults and healthy collegiate athletes in the United States (Howell et al., 2018; Teas and Friedman, 2021). Collectively, mixed results in previous studies have limited researchers to draw a firm conclusion on the association between sleep and gait speed, which requires further investigation.

In addition, previous sensitivity theories have suggested that sex differences on physical performance tend to affect health (Denton et al., 2004). For example, Ruan et al. (2019) found that among female adults the association between poor sleep quality and lower physical function was stronger compared to the male group within a same study. Such a decrease in physical function may have greater influence on sleep among females. Furthermore, one study showed that female with more than 8 h of sleep had a higher risk of sarcopenia compared to the male (Hu et al., 2017). Such findings have implied a sex-specific difference in the relationship between sleep problems and physical function (e.g., gait speed), which deserves a special attention.

As mentioned previously, there has been little attention paid to exploring the association between sleep problems and walking speed, especially among Chinese older adults as a country with largest aging population. In addition, the role of sex difference in this association would be further analyzed. To this end, the present cross-sectional study aimed to investigate the association between sleep problems (sleep quality and sleep duration) and gait speed among Chinese older adults. It is hypothesized that older adults with faster gait speed would have better sleep quality and normal sleep duration, and the faster gait speed would be associated with poor sleep quality and sleep duration in both male and female.

## Materials and Methods

### Design and Sample

A secondary analysis was performed using the Global Aging and Adult Health Survey (SAGE: http://www.who.int/healthinfo/sage/en). The data was conducted from 2007 to 2010 across eight provinces, representing a national sample of populations in China. The detailed survey methods have been described in the previous study (Kowal et al., 2012). In short, a multi-stage cluster sampling method was used to obtain a nationally representative sample in the experimental design. Well-trained interviewers conducted face-to-face household interview surveys (questionnaire) to collect the data. The response rate was 93% in China. The World Health Organization ethical review committee and the Chinese ethics research review board approved the study proposal. All participants agreed with the experiment by signing the informed consent before the study began.

Chinese participants aged 50 years or over were included in the analysis. The participants’ characteristics, gait speed, and sleep problems were extracted from the SAGE database. As a result, 13,367 people were included for analysis in the present study.

### Gait Speed

Gait speed was assessed with the 4 m walking test. Normal speed refers to the participant walks at their usual walking speed; rapid speed refers to the participant walks as fast as they can. The participants were asked to walk at their “normal speed” and “rapid speed.” The time taken in seconds on 4 m walking was recorded. This gait measurement is widely used in previous studies (Gildner et al., 2019; Ruan et al., 2019).

### Sleep Problems

Sleep problems consisted of three questions in the WHO-SAGE survey: sleep quality and sleep duration. For the sleep quality, the question was “Overall in the last 30 days, how much of a problem did you have with sleeping, such as falling asleep, waking up frequently during the night, or waking up too early in the morning?,” and the answers were none, mild, moderate, severe and extreme. Those who responded “severe” and “extreme” were considered to have poor sleep quality. This sleep quality has been used in previous publications using the same survey question on sleep quality (Stranges et al., 2012; Selvamani et al., 2018).

Sleep duration was a measure of the average value of sleep over two nights assessed by using the question: “How many hours did you sleep last night?”. The sleep duration was categorized into <7 h (short sleep duration), 7–8 h (normal sleep duration), and >8 h (longer sleep duration), consistent with previous study (Stranges et al., 2012; Selvamani et al., 2018).

### Covariates

Age, sex, education years, setting (rural or urban), alcohol consumption, smoking (never, current, past), number of chronic diseases (e.g., arthritis, stroke, angina, diabetes), body mass index (BMI), level of physical activity (PA) (Vancampfort et al., 2017) were considered as covariates in the analyses. According to the age, the adults were divided into middle adults (50–64 years old) and older adult (≥65 years old). The total number of chronic diseases was summed for each participant. BMI was calculated by the following equation: $B\,M\,I=\frac{\text{wight}\,\left(\text{kg}\right)}{h\,e\,i\,g\,h\,t^{2}\,\left(m^{2}\right)}$. In line with a previous study, PA was measured using the Global Physical Activity Questionnaire (GPAQ), and the levels of physical activity were classified as ≥150 min/week (meeting the recommended guidelines) and <150 min/week (low PA) (Vancampfort et al., 2017).

### Statistical Analysis

All analyses were conducted with Stata 15.0 (Stata Corp LP, College station, TX, United States), and a *p*-value less than 0.05 (two-tailed) was regarded as significant. All continuous variables were presented as mean and standard deviation (SD), and categorical variables were presented as frequencies. The between group differences were compared by independent *t*-test for continuous variables and chi-squared test for categorical variables. In the full model, multivariable linear regression models were employed to assess the association between gait speed and sleep quality by adjusting for age, sex, education years, marital status, setting, alcohol consumption, smoking, diseases, physical activity, BMI, and sleep duration. Since there was a significant interaction between gait speed and sex in our linear regression models, we estimated the associations between gait speed and sleep problems by stratifying sex. In the models stratified by sex, multivariable linear regression models were employed to assess the association between gait speed and sleep quality by adjusting for age, education years, marital status, setting, alcohol consumption, smoking, diseases, physical activity, BMI, and sleep duration.

## Results

A total of 13,367 participants aged 50 years or over were included in the analysis. The main characteristics of male and female participating in the SAGE study were provided in Table 1. Age did not show a significant difference between male (mean age: 63.23 ± 9.36 years old) and female (mean age: 63.10 ± 9.50 years old) (*p* = 0.22). There were statistically significant differences in the variables between males and females for education years, marital status, BMI, setting, alcohol consumption, smoking, diseases, physical activity, sleep quality, and sleep duration (*p* = 0.04).

**TABLE 1:** Participant characteristics stratified by sex.

Variables		Male (6274)	Female (7093)	*p* value
Age		63.23 ± 9.36	63.10 ± 9.50	0.22
Education (years)		7.53 ± 3.90	6.94 ± 3.89	0.001
BMI		23.66 ± 5.13	24.27 ± 6.05	0.001
Marital	Married/cohabiting	89.21	77.48	0.001
	Never married/separated/divorced/widowed	10.79	22.52	
Setting	Urban	65.89	42.17	0.001
	Rural	34.11	57.83	
Alcohol consumption***	Yes	53.49	10.81	0.001
	No	46.51	89.19	
Smoking	Never	34.38	95.24	0.001
	Current	53.48	3.73	
	Past	12.14	1.03	
Physical activity	≤150 min/week	46.37	49.80	0.001
	>150 min/week	53.63	50.20	
Disease	0	49.59	44.27	0.001
	1–2	39.80	43.94	
	3+	10.62	11.79	
Overall sleep quality	Good	98.33	96.80	0.001
	Poor	1.67	3.20	
Sleep duration	<7 h	35.11	37.26	0.04
	7–8 h	30.40	29.31	
	>8 h	34.49	33.43	

***P < 0.001.

Table 2 presents the multivariable linear regression analysis results on the association between gait speed and sleep problems. In the full model, after adjusting for the age, sex, education years, marital status, setting, alcohol consumption, smoking, diseases, physical activity, BMI, and sleep duration, slower normal gait speed was significantly associated with poor sleep quality (β = 0.356, 95% CI [0.185, 0.527]), and slower normal gait speed was significantly associated with longer sleep duration (>8 h) (β = 0.141, 95% CI [0.078, 0.205]).

**TABLE 2:** Association between normal gait speed and sleep problems estimated by multivariable linear regression.

Variables		Full model^a^	Model stratified by sex^b^	
		Total (95% CI)	Male (95% CI)	Female (95% CI)
Age	Older vs. Middle	0.549 [0.492, 0.606]	0.534 [0.455, 0.612]	0.547 [0.463, 0.631]
Marital	Yes vs. No	−0.315 [−0.392, −0.238]	−0.318 [−0.438, −0.199]	−0.303 [−0.404, −0.203]
Education years		−0.022 [−0.029, −0.016]	−0.018 [−0.028, −0.009]	−0.028 [−0.037, −0.019]
Sex	Male vs. Female	0.104 [0.036, 0.172]	–	–
Smoking	Yes vs. No	0.007 [−0.027, 0.043]	−0.002 [−0.042, 0.038]	0.105 [0.007, 0.203]
Alcohol	Yes vs. No	−0.031 [−0.092, 0.030]	−0.031 [−0.103, 0.041]	−0.043 [−0.160, 0.074]
Physical activity level	High vs. Low	−0.161 [−0.212, −0.110]	−0.165 [−0.237, −0.093]	−0.145 [−0.217, −0.073]
Setting	Urban vs. Rural	0.032 [−0.021, 0.086]	0.085 [0.007, 0.164]	−0.026 [−0.099, 0.047]
Disease		0.164 [0.138, 0.191]	0.163 [0123, 0.202]	0.166 [0.129, 0.203]
BMI		0.007 [0.002, 0.012]	0.009 [0.001, 0.016]	0.006 [−0.001, 0.012]
**Normal walking speed**				
Poor sleep quality	Yes vs. No	0.356 [0.185, 0.527]***	0.627 [0.341, 0.913]***	0.190 [−0.021, 0.403]
Sleep duration	7–8 h	ref	ref	ref
	<7 h	−0.005 [−0.064, 0.054]	0.001 [−0.091, 0.075]	−0.004 [−0.087, 0.079]
	>8 h	0.141 [0.078, 0.205]***	0.210 [0.121, 0.297]***	0.058 [−0.033, 0.150]

*p < 0.05; **p < 0.01; ***p < 0.001.

*^a^The adjusted model included age, sex, education years, setting, alcohol consumption, smoking, number of diseases, physical activity, BMI, and sleep duration.*

*^b^The adjusted model included age, education years, setting, alcohol consumption, smoking, number of diseases, physical activity, BMI, and sleep duration.*

The association between gait speed and sleep problems by sex-stratified analyses showed in Tables 2, 3. In the male, after adjusting for the age, education years, marital status, setting, alcohol consumption, smoking, diseases, physical activity, BMI, and sleep duration, associations were observed between slower normal gait speed and poor sleep quality (β = 0.627, 95% CI [0.341, 0.913]), and longer sleep duration (>8 h) (β = 0.210, 95% CI [0.121, 0.297]). In females, after adjusting for the age, education years, marital status, setting, alcohol consumption, smoking, diseases, physical activity, BMI, and sleep duration, there were no associations between normal gait speed and sleep problems. Moreover, no significant associations between rapid gait speed and sleep problems were overserved (Table 3).

**TABLE 3:** Association between rapid gait speed and sleep problems estimated by multivariable linear regression.

Variables		Full model^a^	Model stratified by sex^b^	
		Total (95% CI)	Male (95% CI)	Female (95% CI)
Age		0.029 [0.025, 0.034]	0.387 [0.266, 0.508]	0.453 [0.340, 0.5661]
Marital	Yes vs. No	0.417 [0.334, 0.500]	−0.337 [−0.522, −0.153]	−0.353 [−0.488, −0.218]
Education years		−0.019 [−0.028, −0.009]	−0.018 [−0.033, −0.003]	−0.020 [−0.032, −0.007]
Sex	Male vs. Female	0.028 [−0.071, 0.127]	–	–
Smoking	Yes vs. No	−0.013 [−0.066, 0.039]	−0.014 [−0.076, 0.048]	−0.002 [−0.134, 0.130]
Alcohol	Yes vs. No	0.151 [−0.239, −0.063]	0.142 [−0.031, 0.251]	0.178 [−0.022, 0.335]
Physical activity level	High vs. Low	−0.165 [−0.239, −0.091]	−0.179 [−0.290, −0.061]	−0.149 [−0.245, −0.052]
Setting	Urban vs. Rural	0.080 [0.002, 0.157]	0.076 [−0.045, 0.197]	0.077 [−0.021, 0.175]
Disease		0.135 [0.096, 0.174]	0.157 [0.097, 0.218]	0.113 [0.063, 0.163]
BMI		0.007 [−0.001, 0.014]	0.009 [−0.003, 0.020]	0.005 [−0.004, 0.013]
Poor sleep quality	Yes vs. No	−0.029 [−0.282, 0.224]	0.088 [−0.366, 0.541]	−0.090 [−0.375, 0.196]
Sleep duration	7–8 h	ref	ref	ref
	<7 h	0.042 [−0.043, 0.128]	0.046 [−0.084, 0.173]	0.039 [−0.071, 0.150]
	>8 h	0.038 [−0.054, 0.131]	0.024 [−0.112, 0.158]	0.059 [−0.064, 0.182]

*p < 0.05; **p < 0.01; ***p < 0.001.

*^a^The adjusted model included age, sex, education years, setting, alcohol consumption, smoking, number of diseases, physical activity, BMI, and sleep duration.*

*^b^The adjusted model included age, education years, setting, alcohol consumption, smoking, number of diseases, physical activity, BMI, and sleep duration.*

## Discussion

The present study investigated the association between gait speed and sleep problems among Chinese adults aged 50 and greater. Our main findings showed that slower normal gait speed was independently associated with poor sleep quality and longer sleep duration (>8 h) after controlling for the covariates (i.e., age, sex, setting, alcohol consumption) among this age group. Moreover, there were negatively significant associations of slower normal gait speed with sleep quality and longer sleep duration (>8 h) in Chinese male adults aged 50 and greater.

The association between sleep quality and gait speed in adults has not been well studied. Previous studies have demonstrated that sleep quality was negatively associated with handgrip strength and gait variability in adults (Hu et al., 2017; Liu et al., 2019; Teas and Friedman, 2021). A real-world gait monitoring study found a direct correlation between sleep disorder and gait speed in patients with rapid eye movement sleep (Del Din et al., 2020). Our findings extend previous literature on the association between sleep quality and gait speed in Chinese adults aged 50 or older. The present study showed that Chinese adults aged 50 years or over with poor sleep quality or longer sleep duration had significantly slower normal walking speed. These findings are consistent with previously published work (Kasović et al., 2021). However, conflicting results on sleep quality and normal walking speed have been reported. Agmon et al. (2016) found that there was no significant association between poor sleep quality and slower walking speed in adults. The different results may be due to the smaller number of people included in the latter study than ours.

With regard to the role of sex differences in the relationship between sleep problems and gait speed, our finding suggested that a significant association of slower gait speed with poor sleep quality and longer sleep duration were only observed among male participants, which was in agreement with previous studies (Goldman et al., 2007; Zhang et al., 2021). Goldman et al. (2007) suggested that compared to adults with normal sleep duration (7–8 h), longer sleep duration in both male and female adults was associated with slower normal walking speed. Another study showed that the link between sleep problems and slower walking speed is apparent in England females and males (Zhang et al., 2021). As there are few studies on exploring the relationship between gait speed and sleep, the reasons for these different results may be the influence of different populations and sample sizes in different regions and sample sizes of populations. Thus, future studies investigating the role of sex differences in sleep and gait speed are necessary.

According to the present study’s findings, poor sleep quality and longer sleep duration were associated with slower normal walking speed in Chinese adults. Some possible reasons might be explained. One explanation may be that slow walking speed is attributed to reduced muscle strength and function. Sleep-related problems can result in imbalance of endocrine secretion associated with skeletal muscle health (e.g., insulin-like growth factor, irisin, and inflammatory response), which possibly disrupts the protein synthesis and increases the protein degradation, thereby limiting the increase in muscle strength (Tan et al., 2020; Zhang et al., 2021). A decline in leg muscle strength can cause poor imbalance and an effective protective response, possibly influencing the walking speed (Zhang et al., 2021). Further, the limitation of physical function (e.g., slow walking speed) may affect the sleep quality in adults (Goldman et al., 2007; Zhang et al., 2021). Other explanations may be that the association between gait speed and sleep was influenced by the deterioration in executive function, which contributes to regulation of the gait control (Watson et al., 2010). At the same time, executive function has been observed to be associated with sleep quality. Sleep disorder causes inefficient executive function and cognitive decline by directly affecting the frontal lobe activation. Dual task is one of the sensitive response paradigms of executive function impairment. The subjects with lower cognitive reserve capacity (e.g., poor executive function) may adapt the “cognitive first” and “posture second” strategy, resulting in gait variability and increased risk of falls (Agmon et al., 2014). In particular, abnormal gait is closely associated with decreased executive function, especially attention, during walking and even dual task (Kirshner et al., 2021). Moreover, there is a significant link between brain structure (e.g., basal ganglia, cerebellum, pontine tegmentum) in areas that they affect the cognitive processes of gait control by interaction between brain stem and cerebral cortex (Takakusaki, 2017), thereby affect the sleep problems (Stefani et al., 2013; Lewis, 2015). Especially for middle-aged and elderly people, changes in brain structure may contribute to reducing sleep quality and gait speed. Additionally, socioemotional factors, including anxiety and depression, are essential responses to human health. These factors contribute to regulating the correlation between sleep problems and age-related declines in physical function such as walking (Ambrose et al., 2013). It was observed that mental health (e.g., depression) could be related to poor sleep quality in adults and also mediates decreased physical function (e.g., gait speed)and sleep problems (Kuralay and Kiyak, 2018; Vega et al., 2019).

There were several limitations to be aware of when interpreting the results of the current study. First, it is difficult to infer a causal relationship between sleep problems and gait speed based on the current cross-sectional study. Second, the 4 m walking is shorter distance, and its validity may be not high degree compared to long distance walking measurements (e.g., 10 m walking test). However, the reliability of 4 m walking test is excellent (Peters et al., 2013). Third, all variables are subjective measures rather than using the sophisticated instrument to assess, which can cause false-positive results because of inevitable measurement errors. Four, subjects were Chinese adults aged 50 years or over, and thus the findings may not extend to all other populations. Future research could benefit from investigating these associations in other age or ethnic populations. Fifth, the present findings were obtained using the dataset from 2007 to 2010. It may influence the generality of the findings to some extent due to the rapid changes in the social living environment.

## Conclusion

The present study suggested that poor sleep quality and longer sleep duration (>8 h) were significantly associated with slower normal walking speed in Chinese adults. It implies that older people should engage in physical activity in their life to improve their sleep quality. Further studies are needed to identify the role of sex differences in association between gait speed and sleep problems.

## Data Availability Statement

The datasets presented in this study can be found in online repositories. The names of the repository/repositories and accession number(s) can be found below: https://apps.who.int/healthinfo/systems/surveydata/index.php/catalog.

## Ethics Statement

This study was reviewed and approved by the World Health Organization ethical review committee and the Chinese ethics research review board approved the study proposal. Written informed consent was obtained from the individual(s) for the publication of any potentially identifiable images or data included in this article.

## Author Contributions

LW and BZ: conceptualization and methodology. LW: formal analysis, data curation, and writing—original draft preparation. BZ: writing—review and editing. Both authors have read and agreed to the published version of the manuscript.

## Conflict of Interest

The authors declare that the research was conducted in the absence of any commercial or financial relationships that could be construed as a potential conflict of interest.

## Publisher’s Note

All claims expressed in this article are solely those of the authors and do not necessarily represent those of their affiliated organizations, or those of the publisher, the editors and the reviewers. Any product that may be evaluated in this article, or claim that may be made by its manufacturer, is not guaranteed or endorsed by the publisher.
